# Cerebrospinal Fluid Leak Secondary to Whiplash Injury

**DOI:** 10.7759/cureus.67860

**Published:** 2024-08-26

**Authors:** Robin Okpara, Kofi Agyare, Daniel Ho, Kirie Psaromatis, George Rodenko

**Affiliations:** 1 Radiology, Texas Tech University Health Sciences Center School of Medicine, Lubbock, USA; 2 Radiology, Medical Center Hospital, Odessa, USA

**Keywords:** neck pain, epidural blood patch, intracranial hypotension, headache, cerebrospinal fluid leak

## Abstract

Although rare, cerebrospinal fluid (CSF) leaks can prove to be detrimental if severe. Usually secondary to dural punctures, CSF leaks can present as severe headaches, neck pain, blurry vision, confusion, and nausea. However, patients can also be asymptomatic. Due to the rarity and variability in symptom presentation, the diagnosis of these leaks is often missed. We present a case of a 15-year-old female who had been experiencing severe headaches after she hyperextended her neck during a horseback ride. On diagnostic imaging, a CT myelogram confirmed a CSF leak with contrast extravasation along the left T9 nerve root up to the T3-T4 levels. After confirmation, the patient received an epidural blood patch, with 15 ml of autologous blood injected into the epidural space. After the procedure, the patient experienced significant symptomatic relief, resulting in an 80% improvement in her pain scale. Our case demonstrates how a prompt and accurate diagnosis of a CSF leak can optimize patient outcomes.

## Introduction

A dural puncture refers to an accidental injury to the dura mater, a membrane that surrounds the fluid that coats the spinal cord [1]. This injury can result in the leakage of cerebrospinal fluid (CSF), leading to various symptoms such as neck pain, headaches, nausea, vomiting, blurry vision, and confusion [2,3]. Post-dural puncture headaches are usually the most common presenting symptom and the reason patients seek medical attention. These headaches are theorized to be caused by decreased CSF pressure secondary to the leakage, leading to vasodilation and increased cerebral blood flow [2]. 

Diagnostic imaging techniques, such as CT myelography or MRI, are typically used to diagnose dural punctures [4]. Treatment typically involves bed rest, administering fluids, and analgesics. If symptoms persist, an epidural blood patch may be necessary to seal the dural puncture and prevent further leakage of CSF. An epidural blood patch is a procedure where a small amount of autologous blood is injected into the epidural space to stop a CSF leak [5]. This case report describes a 15-year-old female patient who presented with a chief complaint of a headache and was found to have a CSF leak in her thoracic spine.

This article was previously presented as a poster at the 2024 Texas Tech University Health Sciences Center Graduate School of Biomedical Sciences (TTUHSC GSBS) Annual Student Research Week on February 28, 2024.

## Case presentation

A 15-year-old female with no significant past medical history presented for an epidural blood patch upon referral from her pediatrician due to concern of a CSF leak. She had been experiencing severe chronic headaches for two weeks that began shortly after an incidence of neck trauma, in which her neck hyperextended during a horseback ride. She reported that the headaches were worse while standing, decreasing in severity while supine. The patient also reported experiencing mild nausea and vomiting.

**Figure 1:**
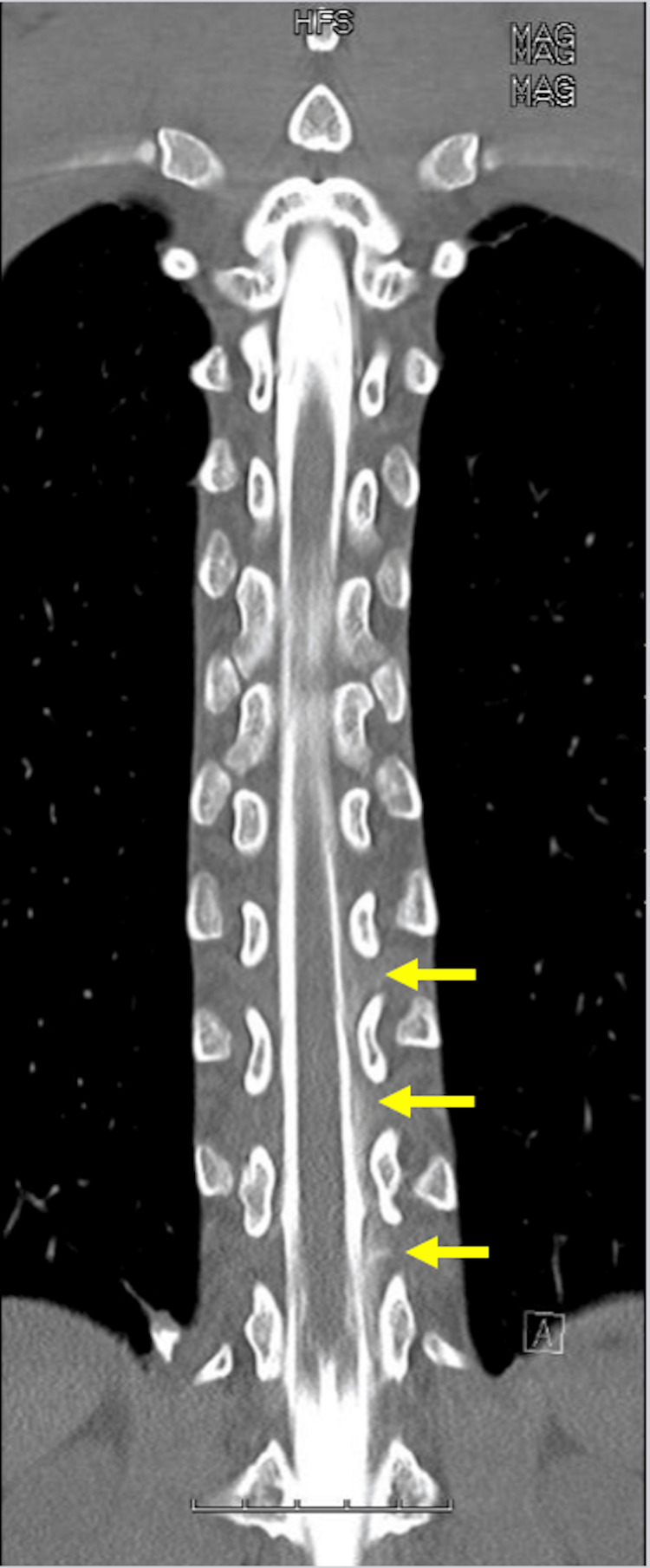
T-spine myelogram image depicts contrast extravasation along several lower nerve roots (yellow arrows)

**Figure 2:**
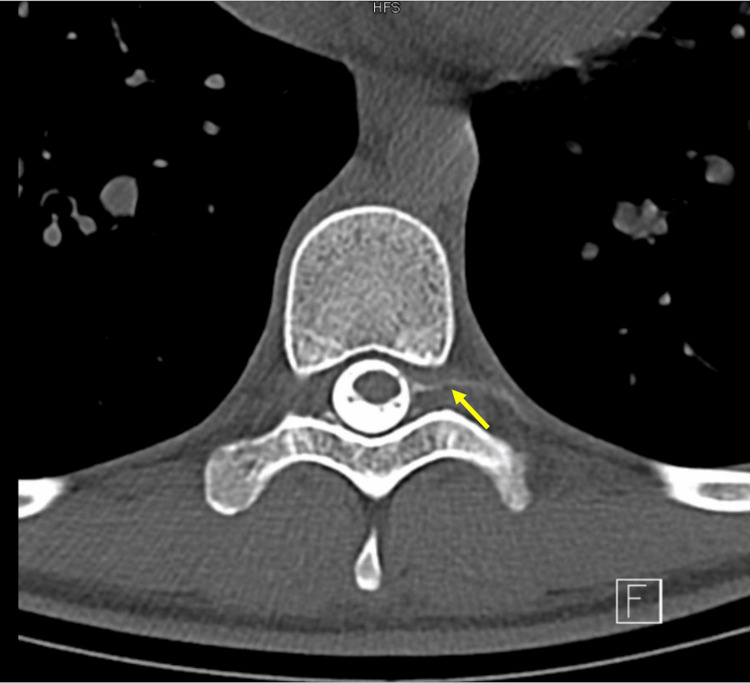
T-spine myelogram image depicts contrast extravasation along the left T9 nerve root, extending to the intervascular region (yellow arrow)

The patient was noted to be hemodynamically stable with normal lab values. Her neurological exam noted no focal deficits, with all other physical exam findings unremarkable. CT myelogram was performed. Thoracic spine images with intrathecal contrast showed significant extrathecal contrast at the lumbar puncture and injection site extending cephalad and along the left lateral margin of the thecal sac, exiting several neural foramina to at least T3-T4 levels (Figures 1-2). 

**Figure 3:**
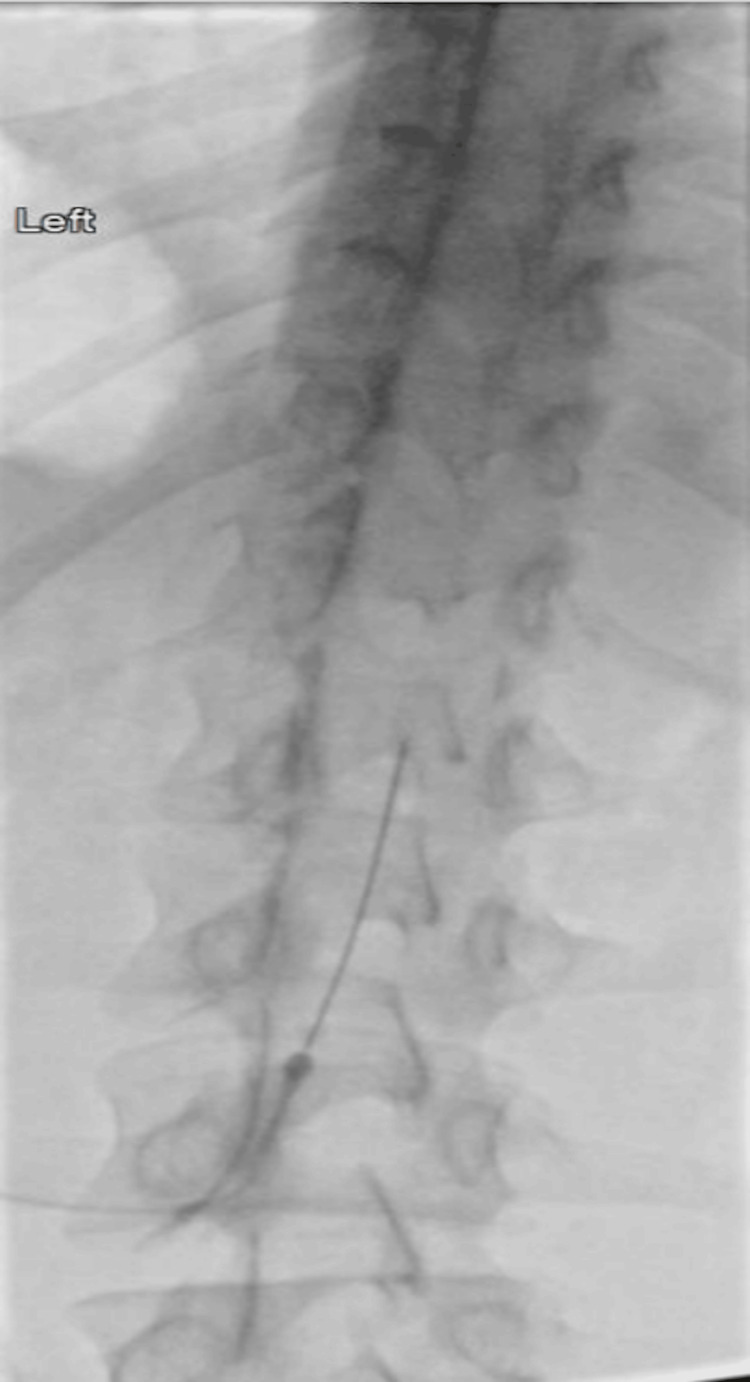
Epidural catheter placed to the left of the midline for blood patch induction

Under fluoroscopic visualization, a 22-gauge spinal needle was advanced through the interspinous ligaments into the dorsal epidural space at the T12-L1 level. Epidural placement of the needle tip was corroborated by an epidural injection of 4.5 mm of Omnipaque 180. A total of 15 mL of autologous blood was injected into the epidural space (Figure 3). The patient experienced significant symptomatic relief after the procedure, resulting in 80% improvement. 

## Discussion

A well-accepted mechanism of dural tears and intracranial hypotension (ICH) includes forceful flexion and extension of the cervical spine [6]. In our patient's case, a CSF leak secondary to forceful flexion of her cervical spine led to ICH, which led to the symptoms she experienced after the injury. CSF leaks are most often seen as a complication of neurological surgical procedures, such as a lumbar puncture, spinal manipulation, or accidental spinal trauma, as in the case of this patient [2]. There are several reported cases of dural tears occurring spontaneously and after traumatic injuries; however, there are less commonly reported cases with minor injuries, such as in this case. Another interesting cause of reported dural tears has been through chiropractic manipulation, which is very uncommon and not well documented [7].

ICH typically presents with an array of symptoms. The most common of these include an orthostatic headache that is relieved when the patient is supine, reported by 97% of patients [8,9]. About 50% of patients have reported auditory disturbances, including dizziness, tinnitus, vertigo, aural fullness, or hyperacusis. These disturbances can be accompanied by nausea and vomiting, as reported in about 50-70% of patients [8]. A smaller percentage of patients have reported ocular symptoms such as blurred vision or photophobia [3]. Cognitive function dysfunctions and cranial nerve symptoms are found in fewer patients [10]. The patient presented in this case study reported symptoms of orthostatic headache, nausea, and vomiting only.

The standard treatment for a CSF leak typically depends on its size, cause, and location [2]. Small leaks have been known to resolve on their own with conservative treatments, including bed rest, increasing fluid intake, elevation of the head of the bed, and avoidance of strenuous activities or exercises [2,9]. Bed rest reduces pressure on the affected areas, allowing the leak to heal over time [2]. Hydration will enable the body to maintain adequate fluid levels, decreasing symptoms. Avoiding exacerbating activities or anything that can cause an increase in pressure, such as coughing, sneezing, Valsalva, or any straining mechanisms, can also reduce symptoms [2]. Patients should take pain medication as needed to relieve headaches or potential neck pain, initially limited to acetaminophen or ibuprofen. Advanced treatment, such as an epidural blood patch, is warranted if the leak persists [9]. Monitoring patients with CSF leaks is crucial to determine any worsening symptoms or leaks that don’t heal independently.

More significant leaks may require advanced intervention. The most common intervention is an epidural blood patch, which was performed on our patient. The procedure involves injecting a small amount of the patient's blood (10 to 55 mL) into the epidural space surrounding the spinal cord, putting pressure on the CSF leak to seal it, preventing further leakage of CSF fluid [5,11]. This procedure is cost-effective, low-risk, and, in many cases, resolves symptoms promptly. Other treatments, such as endoscopic or surgical repair, may be necessary when symptoms are refractory to standard therapy or if symptoms are chronic and persist [2].

## Conclusions

This case illustrates a CSF leak's prompt and accurate diagnosis, leading to an optimal patient outcome. A leak can potentially harm cerebral perfusion and function, increase the risk of direct trauma due to loss of fluid cushion, and potentially create a pathway for life-threatening CNS infections. Recognition of a potential leak is critical to initiating appropriate therapy and preventing severe progressive neurological side effects. The key symptom to look for and monitor is headache, which is typically orthostatic in nature.

## References

[1] Dobrocky T, Nicholson P, Häni L (2022). Spontaneous intracranial hypotension: searching for the CSF leak. Lancet Neurol.

[2] Severson M, Schaurich CG, Strecker-McGraw MK (2024). Cerebrospinal fluid leak. StatPearls.

[3] Jurcau MC, Jurcau A, Hogea VO, Diaconu RG (2024). Spontaneous intracranial hypotension: Case report and update on diagnosis and treatment. Diagnostics (Basel).

[4] Eross EJ, Dodick DW, Nelson KD, Bosch P, Lyons M (2002). Orthostatic headache syndrome with CSF leak secondary to bony pathology of the cervical spine. Cephalalgia.

[5] Tubben RE, Jain S, Murphy PB (2024). Epidural blood patch. StatPearls.

[6] De Gelb D, Lenke L, Pond J (1998). Dural tear associated with a flexion distraction subluxation to the cervical spine without neurologic injury. Acta Orthop Belg.

[7] Mathews MK, Frohman L, Lee HJ, Sergott RC, Savino PJ (2006). Spinal fluid leak after chiropractic manipulation of the cervical spine. Arch Ophthalmol.

[8] D'Antona L, Jaime Merchan MA, Vassiliou A, Watkins LD, Davagnanam I, Toma AK, Matharu MS (2021). Clinical presentation, investigation findings, and treatment outcomes of spontaneous intracranial hypotension syndrome: a systematic review and meta-analysis. JAMA Neurol.

[9] Knutson GA (2006). Intracranial hypotension causing headache and neck pain: a case study. J Manipulative Physiol Ther.

[10] Luetzen N, Dovi-Akue P, Fung C, Beck J, Urbach H (2021). Spontaneous intracranial hypotension: diagnostic and therapeutic workup. Neuroradiology.

[11] Mokri B (2003). Headaches caused by decreased intracranial pressure: diagnosis and management. Curr Opin Neurol.

